# Interactive Panel Summaries of the 2024 Voice AI Symposium

**DOI:** 10.3389/fdgth.2025.1484521

**Published:** 2025-03-27

**Authors:** Jean-Christophe Bélisle-Pipon, James Anibal, Ruth Bahr, Steven Bedrick, Oita Coleman, David Dorr, Barbara J. Evans, Guy Fagherazzi, Alexander Gelbard, Satrajit Ghosh, Anita Ho, Christie Jackson, Dale Joachim, Lampros Kourtis, Andrea Krussel, Amir Lahav, Breanna Leuze, Bob MacDonald, Geralyn Miller, Vivek Mohan, Matthew Naunheim, Maria Powell, Anaïs Rameau, Sat Ramphal, Vardit Ravitsky, Charlie Reavis, Samantha Salvi Cruz, Jamie Toghranegar, Adam Vogel, Stephanie Watts, Joseph Yracheta, Robin Zhao, The Bridge2AI-Voice Consortium, Yael Bensoussan, Yael Bensoussan

**Affiliations:** ^1^ Faculty of Health Sciences, Simon Fraser University, Burnaby, BC, Canada; ^2^ Center for Interventional Oncology, Clinical Center, National Institutes of Health (NIH), Bethesda, MD, United States; ^3^ Computational Health Informatics Lab, Department of Engineering Science, Institute of Biomedical Engineering, University of Oxford, Oxford, United Kingdom; ^4^ Department of Communication Sciences & Disorders, University of South Florida, Tampa, FL, United States; ^5^ Departments of Medical Informatics and Clinical Epidemiology and Medicine, Oregon Health and Science University, Portland, OR, United States; ^6^ Open Voice TrustMark Initiative, Apex, NC, United States; ^7^ Levin College of Law, University of Florida, Gainesville, FL, United States; ^8^ Deep Digital Phenotyping Research Unit, Department of Precision Health, Luxembourg Institute of Health, Strassen, Luxembourg; ^9^ Department of Otolaryngology–Head and Neck Surgery, Vanderbilt University Medical Center, Nashville, TN, United States; ^10^ McGovern Institute for Brain Research, Massachusetts Institute of Technology, MIT, Cambridge, MA, United States; ^11^ Bioethics Program, University of California San Francisco, San Francisco, CA, United States; ^12^ Sonde Health, Boston, MA, United States; ^13^ Alzheimer’s Drug Discovery Foundation, Boston, MA, United States; ^14^ Office of Health Information and Data Science, Washington University in St. Louis, St. Louis, MO, United States; ^15^ SkyMedAI, Boston, MA, United States; ^16^ CareerFoundry, Berlin, Germany; ^17^ Google, Mountain View, CA, United States; ^18^ Microsoft, Redmond, WA, United States; ^19^ SoundHealth, Sunnyvale, CA, United States; ^20^ Massachusetts Eye and Ear, Boston, MA, United States; ^21^ Department of Otolaryngology, Weill Cornell Medicine, New York, NY, United States; ^22^ Maya AI, Tampa, FL, United States; ^23^ School of Public Health, University of Montreal, Montreal, QC, Canada; ^24^ Dysphonia International, Itasca, IL, United States; ^25^ Department of Otolaryngology, University of South Florida, Tampa, FL, United States; ^26^ Redenlab Inc. & The University of Melbourne, Melbourne, VIC, Australia; ^27^ Native Bio-Data Consortium, Eagle Butte, SD, United States

**Keywords:** audiomics, voice biomarkers, voice artificial intelligence, artificial intelligence, ethical AI

## Abstract

The 2024 Voice AI Symposium presented by the Bridge2AI-Voice Consortium, was a 2-day event which took place May 1st-May 2nd in Tampa, FL. The event included four interactive panel sessions, which are summarized here. All four interactive panels featured an innovative format, designed to maximize engagement and facilitate deep discussions. Each panel began with a 45 min segment where moderators posed targeted questions to expert panelists, delving into complex topics within the field of voice AI. This was followed by a 45 min “stakeholder forum,” during which audience members asked questions and engaged in live interactive polls. Interactive polls stimulated meaningful conversation between panelists and attendees, and brought to light diverse viewpoints. Workshops were audio recorded and transcripts were assembled with assistance from generative A.I tools including Whisper Version 7.13.1 for audio transcription and ChatGPT version 4.0 for content summation. Content was then reviewed and edited by authors.

## Introduction

Voice biomarkers have emerged as a type of digital biomarker which can be integrated into large multimodal artificial intelligence/machine learning models for use in disease monitoring and detection (1). Bioacoustic data contains specific features which can be extracted to indicate evidence of certain diseases such as neurological diseases, mood/psychiatric disorders, voice disorders, and cardiorespiratory disorders (2–4). Given that voice data can be easily and affordably collected, it has the potential to change current methods of disease diagnosis, screening and monitoring of health states. Voice biomarker technology is therefore ideally suited for integration into remote monitoring and low-resource healthcare settings. The field of voice biomarkers and voice artificial intelligence is advancing and research is resulting in the development of tools for validation and implementation. The unique nature of this technology, also comes with concerns regarding patient privacy and ethical use of voice data. The Voice AI Symposium featured four panels of experts who engaged in dialogue with attendees to explore these relevant themes on the use of voice AI technology and voice biomarkers in healthcare. Panel topics discussed the current and future state of voice biomarkers, specific use cases of voice biomarker technology, ethical implications of the use of voice AI in healthcare, and discussions of how to build partnerships to further innovation in the field. The panels at the Voice AI Symposium collectively emphasized the transformative potential of voice biomarkers in healthcare, highlighting the need for interdisciplinary collaboration, ethical frameworks, broader standardized datasets, and stakeholder engagement to address challenges while ensuring responsible and trusted implementation of this innovative technology.

### Panel 1: “Voice Biomarkers: The Current State and Future Directions”

**Moderator**:

• **Yael Bensoussan, MD MSc FRCSC**; Assistant Professor of Otolaryngology at USF

**Panelists:**

• **Adam Vogel, PhD**: Professor Speech Neuroscience, The University of Melbourne; CEO of Redenlab Inc.
• **Dale Joachim, PhD**: VP of Speech Science and Research at Sonde Health, Inc.
• **Bob MacDonald, PhD**: Technical Program Manager at Google
• **Geralyn Miller**: Senior Director of Health AI at Microsoft
• **Breanna Leuze**: President of CareerFoundry, Patient who has Idiopathic Subglottic Stenosis

#### Objectives

The first panel at this year's Voice AI symposium delved into the evolving field of voice biomarkers, offering a comprehensive overview of our current position and future trajectories. This session aimed to unpack the significant strides made in utilizing voice as a powerful biomarker for early disease detection, monitoring, and personalized treatment strategies. Experts discussed the latest research findings, innovative technologies, and methodologies that have propelled the field forward. They also explored the challenges faced in terms of accuracy, privacy concerns, and ethical implications of voice data usage. The panel envisioned the future of voice biomarkers in healthcare, including potential breakthroughs and the role of artificial intelligence in enhancing predictive models. The discussion sought to foster a deeper understanding and collaboration among researchers, clinicians, and technologists, to realize the full potential of voice biomarkers in revolutionizing personalized medicine and patient care.

#### Adam Vogel

Dr. Vogel emphasized that personalized healthcare and clinical trials would benefit significantly from voice biomarker technology. He outlined how Project Euphonia was a strong example of speech recognition technology for individuals with atypical speech using developing personalized models to enhance accessibility. The technology has potential to better support people with ALS and other speech-impacting conditions. Dr. Vogel emphasized the necessity of standardized, comprehensive datasets to ensure equitable access to emerging monitoring tools and maintaining a focus on the end user (patients) when considering development.

#### Dale Joachim

Dr. Joachim discussed the work being done at Sonde Health in identifying voice characteristics correlated with respiratory, mental and other health conditions, as well as on-device passive voice sampling and analysis (instead of cued elicitations). He noted the complexities of collecting representative data across various demographics and regions, highlighting their efforts to amass data from four continents. Dr. Joachim acknowledged the challenges of managing coexisting conditions and extracting reliable biomarkers amidst diverse populations, advocating focus on phonation (voice production) as a standardized measurement that could be less impacted by linguistic or cultural factors.

#### Bob MacDonald

Dr. MacDonald addressed the importance of language inclusivity in voice biomarker technology, as English is only one of many languages spoken globally. He explained that using English as a starting point helps establish a framework for future expansion to other languages like Spanish and Hindi. However, he acknowledged the importance of ensuring inclusivity across all languages and dialects to avoid exacerbating existing health disparities.

#### Geralyn Miller

Geralyn Miller shared Microsoft's perspective, emphasizing their emphasis on transparent and ethical data practices. She spoke about the integration of Nuance technology into Microsoft's cloud-based services, providing AI-powered tools that could address challenges in medical imaging and voice analysis. She also advocated for comprehensive transparency notes accompanying AI models, which detail their development and usage to build clinician trust. Microsoft is also working on the ethical implications through the TRAIN initiative, aimed at establishing responsible AI practices in healthcare.

#### Breanna Leuze

Breanna Leuze offered a unique patient perspective on the importance of voice biomarkers, sharing her experiences with subglottic stenosis and how difficult it was to receive a diagnosis and adequate monitoring. She stressed that remote monitoring using voice biomarker technology could drastically improve patients' lives, particularly for those with rare voice conditions. She also highlighted the value of supportive patient advocacy groups in driving research and creating meaningful clinical questions.

#### Stakeholder forum

Audience participation brought forth various concerns, such as the challenges of implementing voice biomarker technology in low-income regions and ensuring diverse data collection. Some advocated for insurance companies to adopt these technologies for accurate longitudinal assessments, while others were concerned about privacy and ethical challenges. Keith Comito raised the possibility of using blockchain technology to ensure data provenance and ethical usage. Generative AI was identified as a breakthrough in voice biomarker technology, as it can produce synthetic data that mimics real-world speech patterns. However, concerns were raised about the potential misuse of synthetic speech, especially in privacy-sensitive patient populations.

#### Conclusion

Overall, the panel underscored the need for greater collaboration among healthcare professionals, tech developers, researchers, and patients to ensure that voice biomarker technology is effectively implemented, equitable, and trusted. They called for broader datasets, clearer ethical guidelines, and improved patient education to pave the way for the next generation of voice-based diagnostic tools.

### Panel 2: “Diving Deeper with Specific Disease Case Studies”

**Moderator:**

• **Anaïs Rameau, MD, MPhil, MS, FACS**: Assistant Professor of Laryngology, Chief of Dysphagia, and Director of New Technologies, Department of Otolaryngology—Head and Neck Surgery, Weill Cornell Medicine

**Panelists:**

• **Lampros Kourtis, PhD**: SpeechDx Program, Alzheimer's Drug Discovery Foundation, Manager at Gates Ventures
• **Matthew Naunheim, M.D., MBA**: Assistant Professor in Otolaryngology–Head and Neck Surgery
• **Vivek Mohan, MSEE, BS**: VP at SoundHealth
• **James Anibal:** DPhil candidate at the University of Oxford and NIH Clinical Center, NIH-Oxford Scholar

#### Objectives

This panel discussion focused on the innovative intersection of voice biomarkers and artificial intelligence (AI) in the diagnosis and monitoring of specific disease categories, underpinned by real-world case studies. The session aimed to shed light on how cutting-edge research and technology were being applied to detect and manage specific diseases. Panelists presented case studies that illustrated the practical applications, successes, and challenges faced in integrating voice biomarkers with AI algorithms for early detection, ongoing monitoring, and personalized treatment plans. The discussion focused on providing insights into the scientific advancements, the potential for transforming patient care, and the future directions of this promising field. The goal of the panel was for attendees to gain an understanding of the tangible benefits that voice biomarker technologies bring to different disease categories, fostering a deeper appreciation of the role of AI in enhancing diagnostic precision and treatment outcomes.

#### Lampros Kourtis

Dr. Kourtis, program manager of SpeechDX program at the Alzheimer's Drug Discovery Foundation, elaborated on his initiative to compile extensive datasets that correlate voice recordings with clinical data. These datasets are pivotal for the development of algorithms capable of detecting Alzheimer's disease well before the clinical symptoms manifest. Dr. Kourtis stressed the significance of this preemptive approach, noting that such early detection could potentially delay or even prevent the onset of Alzheimer's symptoms through timely intervention. He envisioned a future where routine conversations captured by ubiquitous devices could serve as continuous, passive screenings for early signs of cognitive decline.

#### Matthew Naunheim

Dr. Naunheim, a laryngologist affiliated with Harvard Medical School and Massachusetts Eye and Ear, shared his experiences with the integration of AI in the field of voice medicine. He detailed his current projects which focus on automating the tracking of vocal fold movements and employing voice biomarkers to enhance diagnostic precision for voice disorders. Dr. Naunheim discussed the specific challenges inherent in diagnosing voice and swallowing disorders, which often present with subtle symptoms that are easily overlooked or misdiagnosed. By harnessing AI, he aims to develop more reliable diagnostic tools that can aid clinicians in identifying underlying conditions more accurately and swiftly, thereby improving treatment outcomes.

#### Vivek Mohan

Vivek Mohan from SoundHealth brought a unique perspective on how voice AI can impact treatment modalities. He introduced a novel medical device, coupled with a smartphone application, that employs acoustic resonance therapy to treat nasal congestion. He explained how individual voice biomarkers are used to personalize the treatment, adjusting the therapy's parameters to the user's specific condition. This approach not only enhances efficacy but also user adherence by simplifying the treatment process. He also recounted his experiences with the FDA approval process, highlighting the complexities and challenges faced when introducing new AI-driven medical technologies to the market.

#### James Anibal

James Anibal discussed the application of voice AI technologies in resource-poor settings, such as rural Vietnam, a critical area given the global disparities in healthcare access. His work, positioned at the intersection of academic research and public health implementation, focuses on infectious disease management through voice data. Anibal described how voice-based AI tools are being deployed in regions lacking robust healthcare infrastructures, offering low-cost, scalable solutions for disease screening and monitoring. He emphasized the importance of developing reliable, unbiased AI systems that can operate across different languages and dialects to ensure broad applicability and effectiveness.

#### Stakeholder forum

The panel discussion opened with a question about which disease categories would benefit most from voice biomarkers in the coming years. The options included neurological disorders, mental health issues, cardiovascular disorders, voice and speech disorders, and acute illnesses. Voice and speech disorders emerged as the clear winner, aligning with the interests of many panelists, including Matt, who noted the direct impact of these disorders on voice, making them a straightforward case for the application of voice biomarkers.

Anibal, who works with acute illnesses, shared the challenges and potential in capturing the complex symptoms that often present similarly across different acute conditions. He highlighted the urgency of developing responsive technologies, especially in the face of health crises like pandemics, where rapid data collection from widespread populations is crucial.

Dr. Rameau raised questions about the practical difficulties of collecting data in hospital settings, where noise and patient conditions can complicate data collection. This led to discussions among other panelists about the varying success they've had in similar environments. Julia Hoxha, from Zana in Germany, contrasted this by sharing positive experiences with data collection in hospitals for heart failure and COPD patients, emphasizing the success of co-developing tools with patient groups to enhance usability and compliance. The session also delved into the technical challenges of collecting clean, useful voice data in natural settings. Issues such as noise cancellation, which can also strip useful nuances from voice data, were discussed, highlighting the delicate balance needed in designing data collection methodologies that preserve the richness and authenticity of voice samples.

Panelists further explored the implications of voice data collection beyond the immediate medical applications, discussing the broader societal and ethical considerations, including consent and data privacy. The conversation underscored the need for responsible governance and the development of technologies that respect patient autonomy and privacy while harnessing the potential of voice biomarkers to revolutionize diagnostics and treatment across various health conditions.

The Q&A session reflected a vibrant exchange of ideas, concerns, and insights, fostering a collaborative atmosphere where diverse expertise converged to push the boundaries of what can be achieved with voice technology in healthcare. The discussion highlighted the ongoing challenges and opportunities, emphasizing the importance of multidisciplinary collaboration and ethical consideration in the rapidly evolving field of voice biomarker research.

#### Conclusion

Throughout the panel, the speakers highlighted several overarching themes, including the importance of interdisciplinary collaboration, the challenges of ensuring privacy and data security, and the regulatory hurdles associated with new AI technologies in healthcare. They collectively painted a picture of a future where voice biomarkers could fundamentally alter the landscape of disease diagnosis, monitoring, and management, making healthcare more proactive, personalized, and accessible. The discussion not only illuminated the current state of voice biomarker research but also charted a course for its future development, emphasizing the need for continued innovation and ethical considerations as this exciting field evolves.

### Panel 3: “Trust in the Context of Rapidly Evolving Technology”

**Moderator:**

• **Vardit Ravitsky, PhD**: President and CEO of The Hastings Center

**Panelists:**

• **Anita Ho, PhD, MPH**: Associate Professor at the UCSF Bioethics Program and Centre for Applied Ethics/School of Population and Public Health at the University of British Columbia, Division Vice President of Ethics (Northern California) for CommonSpirit Health, and a Scientist at the Centre for Advancing Health Outcomes
• **Barbara J. Evans, JD, PhD**: Professor of Law and Stephen C. O'Connell Chair, University of Florida Levin College of Law Professor of Engineering and Renwick Faculty Fellow in AI & Ethics, University of Florida, Wertheim College of Engineering
• **Joseph Yracheta, MS:** Native BioData Consortium and Johns Hopkins School of Public Health, dept of Environmental Health Sciences and Engineering
• **Oita Coleman, BS:** head of the Open Voice TrustMark Initiative, leads the global Linux Foundation project

#### Objectives

The third interactive panel revolved around the crucial issue of trust in the rapidly evolving landscape of voice AI technology within healthcare. As these technologies advance at an unprecedented pace, they promise significant enhancements in patient care, diagnostics, and treatment processes. However, this evolution has also brought forth complex challenges related to privacy, data security, ethical considerations, and the potential for biases. The purpose of the discussion was to bring together leading experts from the fields of healthcare, technology, ethics, and law to explore and debate the multifaceted aspects of trust. Panelists addressed how trust could be built and maintained between patients, healthcare providers, and technology developers; the role of transparency, consent, and data protection in fostering trust; and the measures needed to ensure that voice AI technologies were developed and deployed in a manner that is ethical, equitable, and free from biases. Through this discourse, the panel sought to highlight strategies for balancing innovation with the imperative of earning and sustaining the trust of all stakeholders in the healthcare ecosystem.

#### Barbara J. Evans

Dr. Evans opened the panel with a strong critique of traditional privacy protections, such as informed consent, which she argued are insufficient in the digital age. Evans proposed that privacy protection should transition from a rights-based approach to a duty-based approach. This would entail organizations implementing stronger technical safeguards and rigorous contractual obligations. She advocated for active regulation enforcement and suggested that actual privacy protection requires more than just polite consent practices, but a robust framework that adapts to the complexities of modern data environments.

#### Joseph Yracheta

Representing the Native Biodata Consortium, Joseph Yracheta emphasized the importance of indigenous data sovereignty. He discussed how their data governance model is deeply embedded within the legal and cultural frameworks of the Cheyenne River Lakota Nation, highlighting a system that allows for significant control over data by both individuals and the tribe collectively. Yracheta stressed the significance of constructing consent and governance systems that respect cultural values and ensure ethical handling of data, advocating for models that prioritize community involvement and rights over data. He also emphasized that tribes are a legally recognized entity with legal and treaty agreements embedded in US Law and the Constitution, but universities and Federal funders choose to ignore these laws.

#### Oita Coleman

Oita Coleman presented her work with the Open Voice Trustmark initiative, which aims to establish open standards and best practices for voice AI. She focused on the need for an ethical framework that aligns with global legal standards and safeguards privacy while minimizing risks. Coleman highlighted the crucial roles of education and advocacy in promoting ethical practices in voice AI development and deployment. She stressed the importance of collaborative efforts across sectors to ensure that voice AI technologies are developed responsibly and ethically.

#### Anita Ho

Dr. Ho discussed the challenges that arise when commercial interests dominate the development and control of voice AI technologies. She pointed out the potential conflicts between commercial and public interests, especially in terms of data usage that extends beyond the scope of initial patient consent. Dr. Ho called for greater community engagement and a continuous patient input process throughout the AI development lifecycle to ensure technologies are developed that genuinely benefit users and respect their rights and privacy.

#### Stakeholder forum

During the question-and-answer stakeholder forum, a nuanced discussion unfolded around the challenges and nuances of using voice data in research, particularly honing in on consent, data protection, and the tension between commercial and state-funded research endeavors. Participants raised concerns about the current practices of obtaining consent, highlighting that consent forms are often cloaked in complex legal jargon that is difficult for most people to understand. This complexity not only alienates participants but also raises ethical questions about the validity of such consents. A pivotal point of discussion centered on the difficulty of truly anonymizing voice data. Concerns were voiced about how data, collected under simpler consent forms, could potentially be used years later for purposes like voice cloning, thus breaching initial consent agreements. The implications of such uses were debated, particularly in terms of privacy and ethical boundaries.

The forum also delved into the dichotomy between private and public research funding. While acknowledging that commercial entities often accelerate innovation and bring products to market more efficiently, there was a consensus on the need for stringent governance to ensure these advancements do not compromise individual privacy. Participants expressed a greater trust in state-funded institutions, such as the NHS, which they perceived to have stronger ethical oversight and a non-commercial focus that prioritizes public welfare over profit.

Legal and ethical frameworks were discussed as participants called for updated guidelines that could keep pace with the rapid advancements in AI and voice data technology. The enforcement of privacy protections and accountability in the event of data breaches was underscored as a critical need. The conversation also touched on compensation for data usage, where views were mixed. Some advocated for monetary compensation for individuals whose data is used, while others argued that the focus should be on ensuring ethical use and societal benefits from such data.

Technological solutions like blockchain were proposed as means to enhance data security and transparency, potentially giving individuals greater control over their data. This led to suggestions for more inclusive and understandable consent processes, possibly leveraging technology to ensure participants are truly informed and can easily withdraw consent if desired.

Overall, the forum illuminated the complex interplay between ethics, technology, and governance in the use of voice data in research, with a strong emphasis on improving practices to prioritize participant understanding, consent, and the ethical use of data. The discussions painted a picture of a community poised on the brink of technological advancement, yet cautiously navigating the ethical ramifications tied to these developments.

#### Conclusion

The session concluded with a consensus on the need for a multidisciplinary approach to address the ethical challenges presented by voice AI. The panelists emphasized the importance of greater stakeholder engagement, including patients, technologists, ethicists, and legal experts, to ensure the responsible and equitable development of voice AI technologies. Each speaker's contribution highlighted different aspects of the ethical landscape, illustrating the complex interplay between technology, law, culture, and commerce in the field of voice AI.

### Panel 4: “Breaking Silos and Establishing Partnerships to Maximize Impact”

**Moderators:**

• **David Dorr, M.D., M.S.:** OHSU's Chief Research Information Officer, Internal Medicine Doctor
• **Satrajit Ghosh, PhD:** Principal Research Scientist, MIT McGovern Institute

**Panelists:**

• **Amir Lahav, ScD**: Strategic Advisor Clinical Neuroscientist, and Global Thought Leader
• **Alexander Gelbard, MD**: Otolaryngologist, Vanderbilt University Medical Center
• **Charlie Reavis**: President of Dysphonia International
• **Sat Ramphal**: CEO of Co-Founder of Maya AI
• **Guy Fagherazzi, PhD, ADR**: Director of Department of Precision Health, Deep Digital Phenotyping Research Unit, Luxembourg Institute of Health

#### Objectives

The purpose of the fourth and final interactive panel was to underscore the importance of dismantling traditional barriers between disciplines and sectors to foster interdisciplinary innovation across different types of institutions. Panelists shared insights and experiences on forming successful partnerships, navigating the challenges of interdisciplinary collaboration, and leveraging collective strengths to address complex problems. The discussion highlighted case studies of collaboration between tech companies, startups, academic institutions, and healthcare organizations. Through this focused discourse, the panel aimed to shed light on effective strategies for fostering innovation, bridging the gap between industry and academia, and ensuring that the advancements in voice AI were both scientifically rigorous and market-relevant.

#### Amir Lahav

Dr. Lahav discussed his transition from academia to independent consultancy in digital health. He emphasized the importance of collaborative work across pharmaceutical companies, healthcare providers, and startups to leverage AI for improved health outcomes. Dr. Lahav shared insights from his experience in promoting digital innovation and partnerships that aim to leave a healthier world for future generations.

#### Alexander Gelbard

Dr. Alexander Gelbard, an otolaryngologist, focused on his work involving the use of remote data capture of respiratory biomarkers in clinical trials. He detailed how these biomarkers have been crucial for advancing patient care by facilitating early detection and treatment of diseases. Dr. Gelbard highlighted the necessity of a collaborative network of institutions to enhance the adoption of these clinical innovations.

#### Charlie Reavis

Charlie Reavis, President of Dysphonia International, spoke about his organization's role in supporting individuals with voice conditions through education, awareness, and research. He shared personal experiences to underline the impact of these conditions on quality of life and stressed the importance of early diagnosis and treatment, facilitated through educational outreach to healthcare professionals and the public.

#### Sat Ramphal

CEO of Maya AI, Sat Ramphal detailed how his company assists in bringing treatments, medical devices, and drugs to market more efficiently. He explained Maya AI's role in streamlining processes post-clinical trials and ensuring rapid response during product recalls, thereby safeguarding public health. Ramphal emphasized the significance of partnerships in enhancing product development and market access.

#### Guy Fagherazzi

Dr. Fagherazzi introduced his role at the Luxembourg Institute of Health, focusing on the development of digital and vocal biomarkers for various health outcomes. He discussed the international vocal biomarker screening platform, Colive Voice, which aims to standardize the use of vocal biomarkers across multiple languages and continents. Dr. Fagherazzi highlighted the importance of integrating patient and clinician input from the outset to ensure the practical application of research findings.

#### Stakeholder forum

During the question-and-answer stakeholder forum, participants actively engaged with a variety of topics centered around the innovative use of voice technology and partnerships. One notable question involved exploring unusual partnerships for data collection and application beyond the typical academic and industry collaborations. For instance, a speaker discussed how Verizon employees participated in voice recording as part of their employee engagement activities, which was an innovative approach to data gathering outside traditional clinical settings.

Another participant suggested reaching out to microphone manufacturers as they are directly involved in enhancing voice capture technology, which could lead to improved data quality. This suggestion highlighted the potential for partnerships that are not immediately apparent but can offer significant technological advancements.

The discussion also touched on integrating multiple digital technologies for patient monitoring in a clinical trial for ALS. This integration aimed to simplify the patient's interaction with the technology, reducing the burden by consolidating multiple apps into a single, more manageable interface.

A critical point raised was the need to consider voice as a complex and unique identifier, much like a fingerprint, which presents challenges in standardizing voice data analysis. This complexity calls for sophisticated analytical approaches and highlights the potential risks of oversimplification.

Finally, the dialogue also covered the challenges of rapid and effective data sharing, the necessity for ethical frameworks to protect patient data, and the importance of cross-institutional partnerships to enhance resource sharing and broaden the impact of research initiatives. These discussions underscored the multifaceted challenges and opportunities in harnessing voice technology for health and research purposes.

#### Conclusion

The panel discussion concluded with a broad question about the future of AI in health innovation, where each panelist contributed perspectives on how voice AI could significantly impact human health. The session underscored the value of multi-disciplinary approaches and the ongoing need for ethical considerations, particularly in terms of data privacy and the responsible use of AI technologies.

## Summary

Panel discussions of the 2024 Voice AI Symposium consisted of expert discussions and stakeholder forums which engaged attendees in dynamic discussions through interactive polls. Experts discussed the current state of the field and voice biomarker applications for specific diseases such as Alzheimer's Disease, and detection of respiratory disorders like airway stenosis. Ethical considerations for the use of voice AI in medicine were also discussed. Expert panelists suggested that in order to address current challenges such as bias mitigation and scalability of models there is a need for large, diverse, and standardized datasets. Discussions between panelists and attendees reflected the need for community engagement and education in order to understand how to ethically meet the needs of patients. Panelists also discussed how to translate expertise into action by fostering cross-discipline collaborations and exploring diverse funding sources to break silos and foster innovation.

In conclusion, as the field of voice biomarker research evolves, ethical considerations and the need for AI-ready datasets remain at the forefront. Stakeholder engagement, including input from patients and legal experts, will be vital in ensuring the responsible development and deployment of voice AI technologies. Voice biomarkers have great potential to improve the state of disease diagnosis in healthcare, offering new opportunities for proactive, personalized, and accessible tools for disease diagnosis and monitoring. Panelists underscored the critical importance of collaboration across healthcare professionals, AI researchers, ethicists, and patients to navigate the complexities of ethical implementation of voice AI technology.
