# Supplementary material for: Interactive Panel Summaries of the 2024 Voice AI Symposium
Source: Front Digit Health. 2025 Mar 27;7:1484521. doi: 10.3389/fdgth.2025.1484521 (PMC11983451; doi:10.3389/fdgth.2025.1484521)
Supplement: Supplementary file 1 [file Supplementaryfile1.pdf]

The Bridge2AI-Voice Consortium

Yael Bensoussan, University of South Florida, Tampa, FL, US; Olivier Elemento, Weill Cornell Medicine, New York, NY, USA; Anais Rameau, Weill Cornell Medicine, New York, NY, USA; Alexandros Sigaras, Weill Cornell Medicine, New York, NY, USA; Satrajit Ghosh, Massachusetts Institute of Technology, Boston, MA, USA; Maria Powell, Vanderbilt University Medical Center, Nashville, TN, USA; Vardit Ravitsky, University of Montreal, Montreal, Quebec, Canada; Jean-Christophe Bélisle-Pipon, Simon Fraser University, Burnaby, BC, Canada; David Dorr, Oregon Health & Science University, Portland, OR, USA; Phillip Payne, Washington University in St. Louis, St. Louis, MO, USA; Alistair Johnson, University of Toronto, Toronto, Ontario, Canada; Ruth Bahr, University of South Florida, Tampa, FL, USA; Donald Bolser, University of Florida, Gainesville, FL, USA; Frank Rudzicz, Dalhousie University, Halifax, NS, Canada; Jordan Lerner Ellis, University of Toronto, Toronto, ON, Canada; Kathy Jenkins, Boston Children's Hospital, Boston, MA, USA; Shaheen Awan, University of Central Florida, Orlando, FL, USA; Micah Boyer, University of South Florida, Tampa, FL, USA; Bill Hersh, Oregon Health & Science University, Portland, OR, USA; Andrea Krussel, Washington University in St. Louis, St. Louis, MO, USA; Steven Bedrick, Oregon Health & Science University, Portland, OR, USA; Toufeeq Ahmed Syed, UT Health, Houston, TX, USA; Jamie Toghranegar, University of South Florida, Tampa, FL, USA; James Anibal, University of South Florida, Tampa, FL, USA; Duncan Sutherland, New York, NY, USA; Enrique Diaz-Ocampo, University of South Florida, Tampa, FL, USA; Elizabeth Silberhoz, University of South Florida, Tampa, FL, USA; John Costello, Boston Children's Hospital, Boston, MA, USA; Alexander Gelbard, Vanderbilt University Medical Center, Nashville, TN, USA; Kimberly Vinson, Vanderbilt University Medical Center, Nashville, TN, USA; Tempestt Neal, University of South Florida, Tampa, FL, USA; Lochana Jayachandran, Mt. Sinai Health, Toronto, ON, Canada; Evan Ng, The Hospital for Sick Children, Toronto, ON, Canada; Selina Casalino, Mt. Sinai Health, Toronto, ON, Canada; Yassmeen Abdel-Aty, University of South Florida, Tampa, FL, USA; Karim Hanna, University of South Florida, Tampa, FL, USA; Theresa Zesiewicz, University of South Florida, Tampa, FL, USA; Elijah Moothedan, Florida Atlantic University, Boca Raton, FL, USA; Emily Evangelista, University of South Florida, Tampa, FL, USA; Samantha Salvi Cruz, Vanderbilt University Medical Center, Nashville, TN, USA; Robin Zhao, Weill Cornell Medicine, New York, NY, USA; Mohamed Ebraheem, University of South Florida, Tampa, FL, USA; Karlee Newberry, University of South Florida, Tampa, FL, USA; Iris De Santiago, University of South Florida, Tampa, FL, USA; Ellie Eiseman, University of South Florida, Tampa, FL, USA; JM Rahman, University of South Florida, Tampa, FL, USA; Stacy Jo, Boston Children's Hospital, Boston, MA, USA; Anna Goldenberg, Hospital for Sick Children, Toronto, ON, Canada
