# Supplementary figures and images for: Interactive Panel Summaries of the 2024 Voice AI Symposium
Source: Front Digit Health. 2025 Mar 27;7:1484521. doi: 10.3389/fdgth.2025.1484521 (PMC11983451; doi:10.3389/fdgth.2025.1484521)

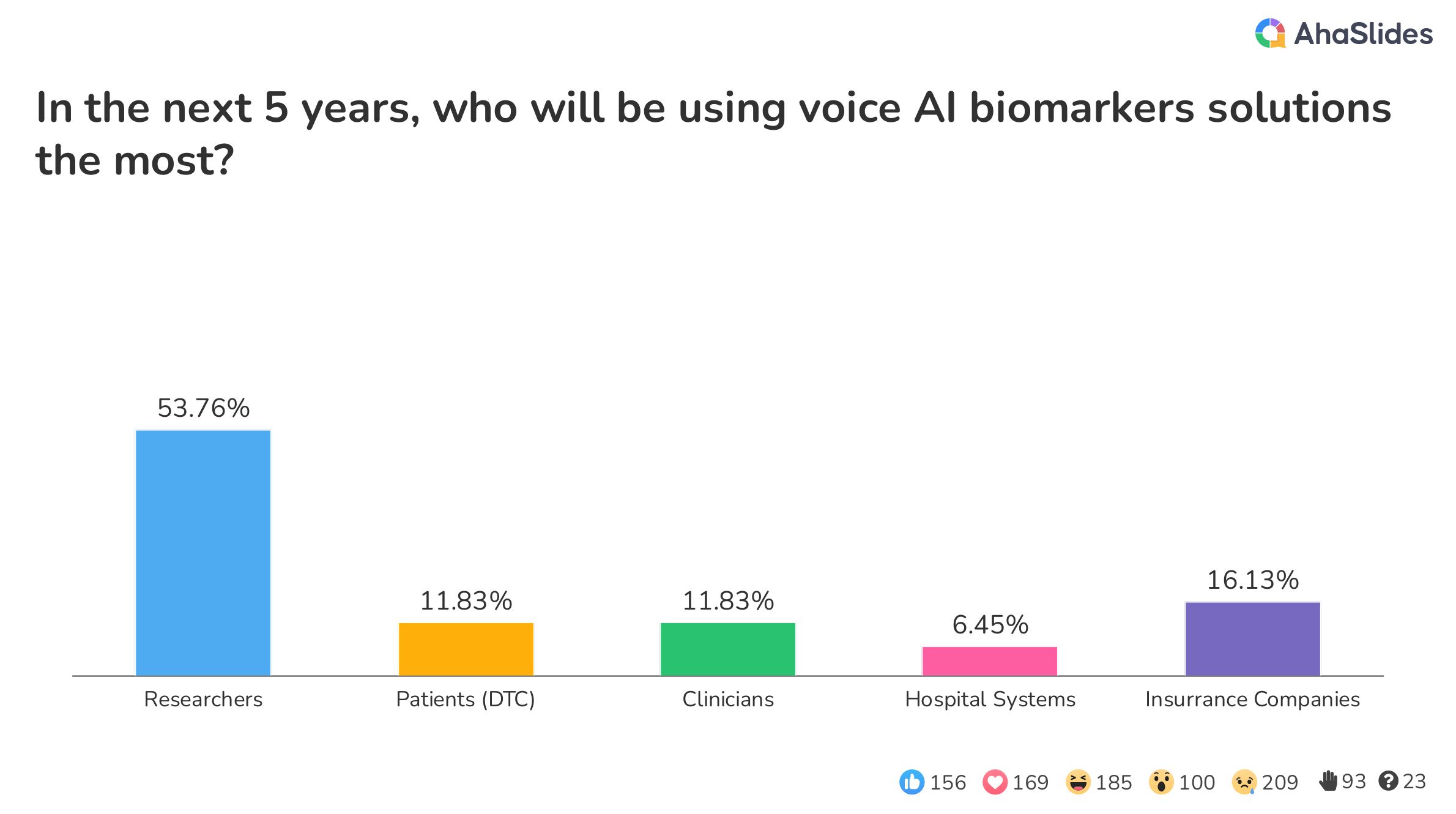

Supplement: Supplementary file 2 [file Image1.jpeg]

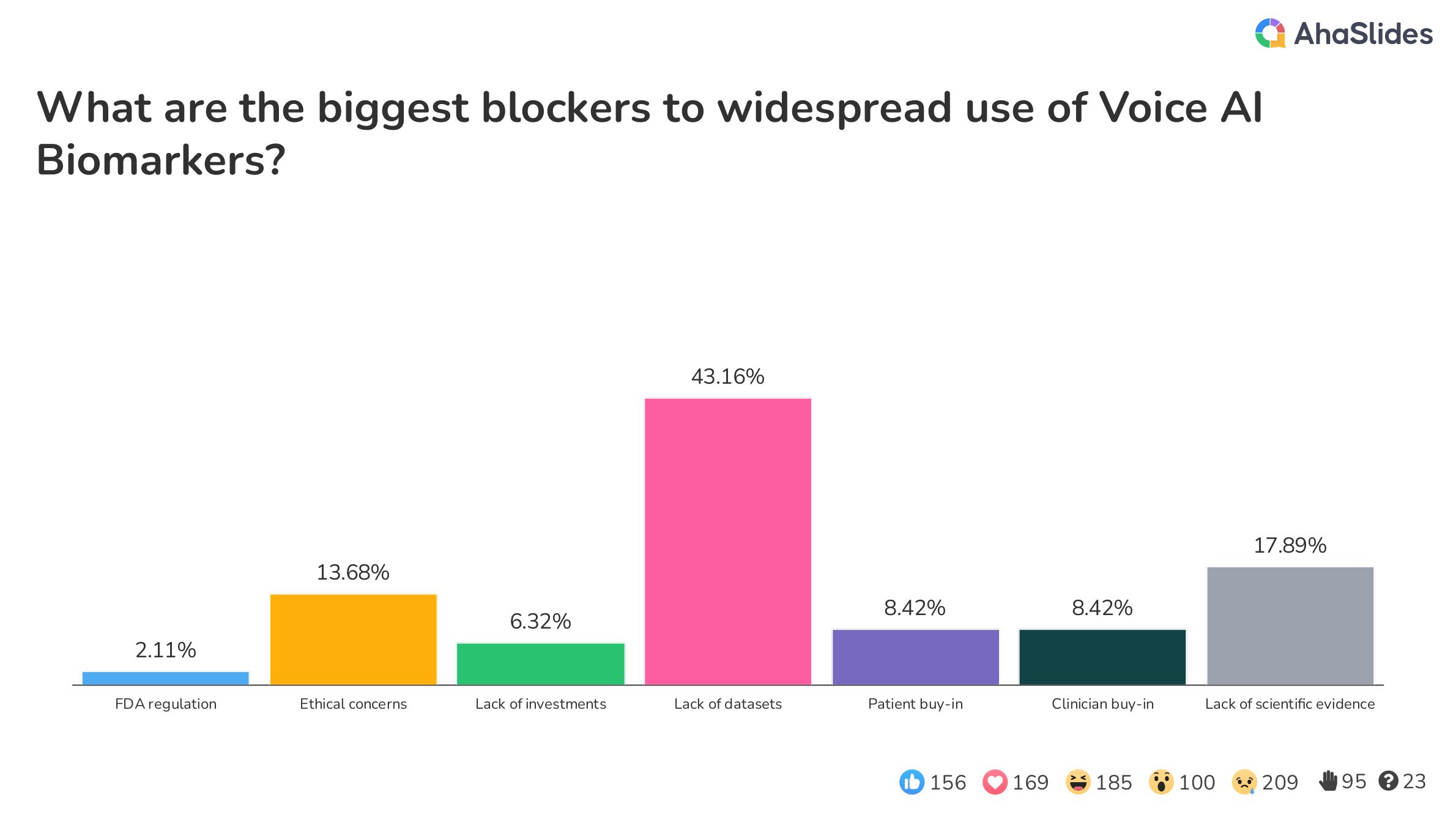

Supplement: Supplementary file 3 [file Image2.jpeg]

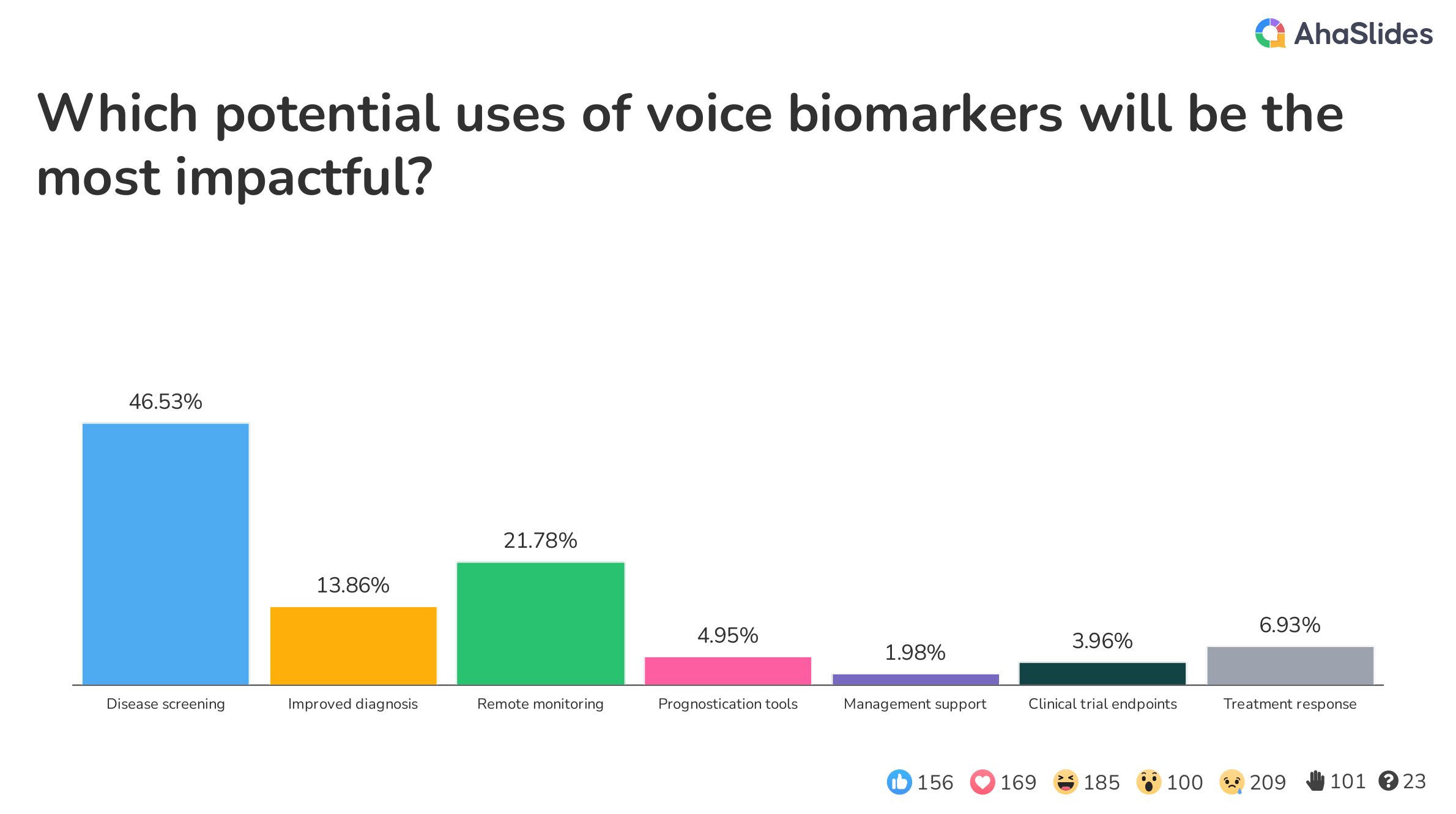

Supplement: Supplementary file 4 [file Image3.jpeg]

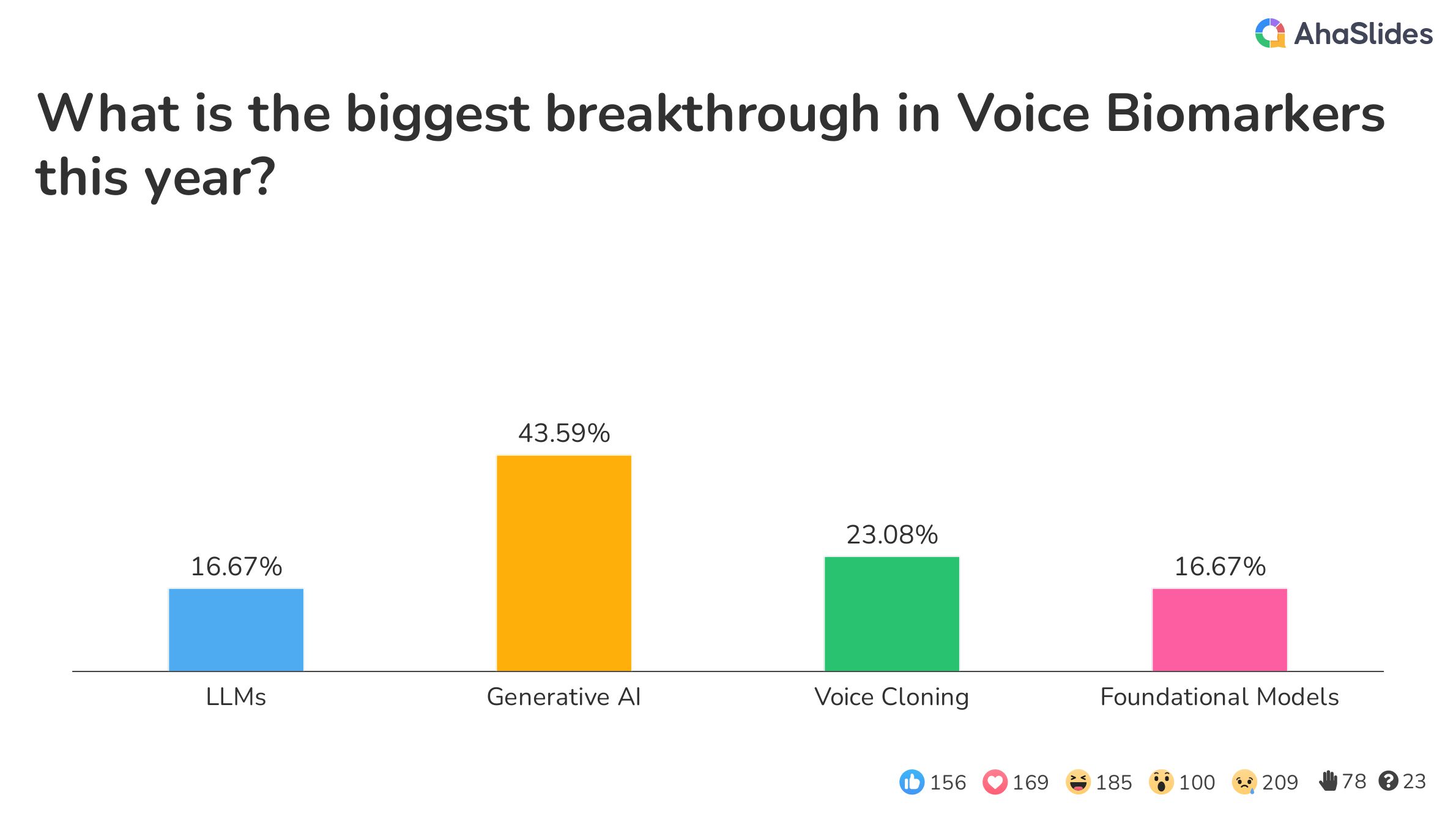

Supplement: Supplementary file 5 [file Image4.jpeg]

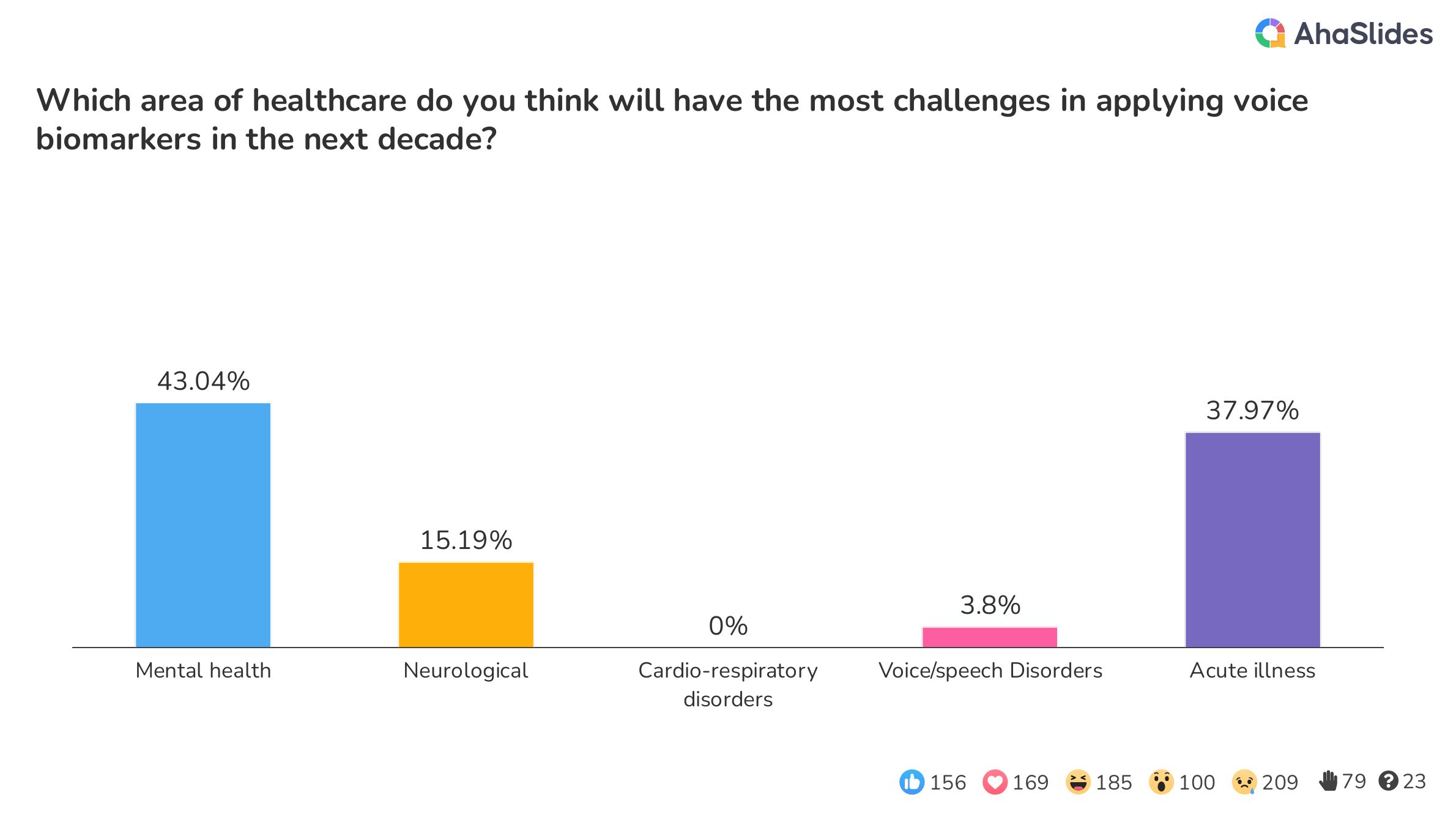

Supplement: Supplementary file 6 [file Image5.jpeg]

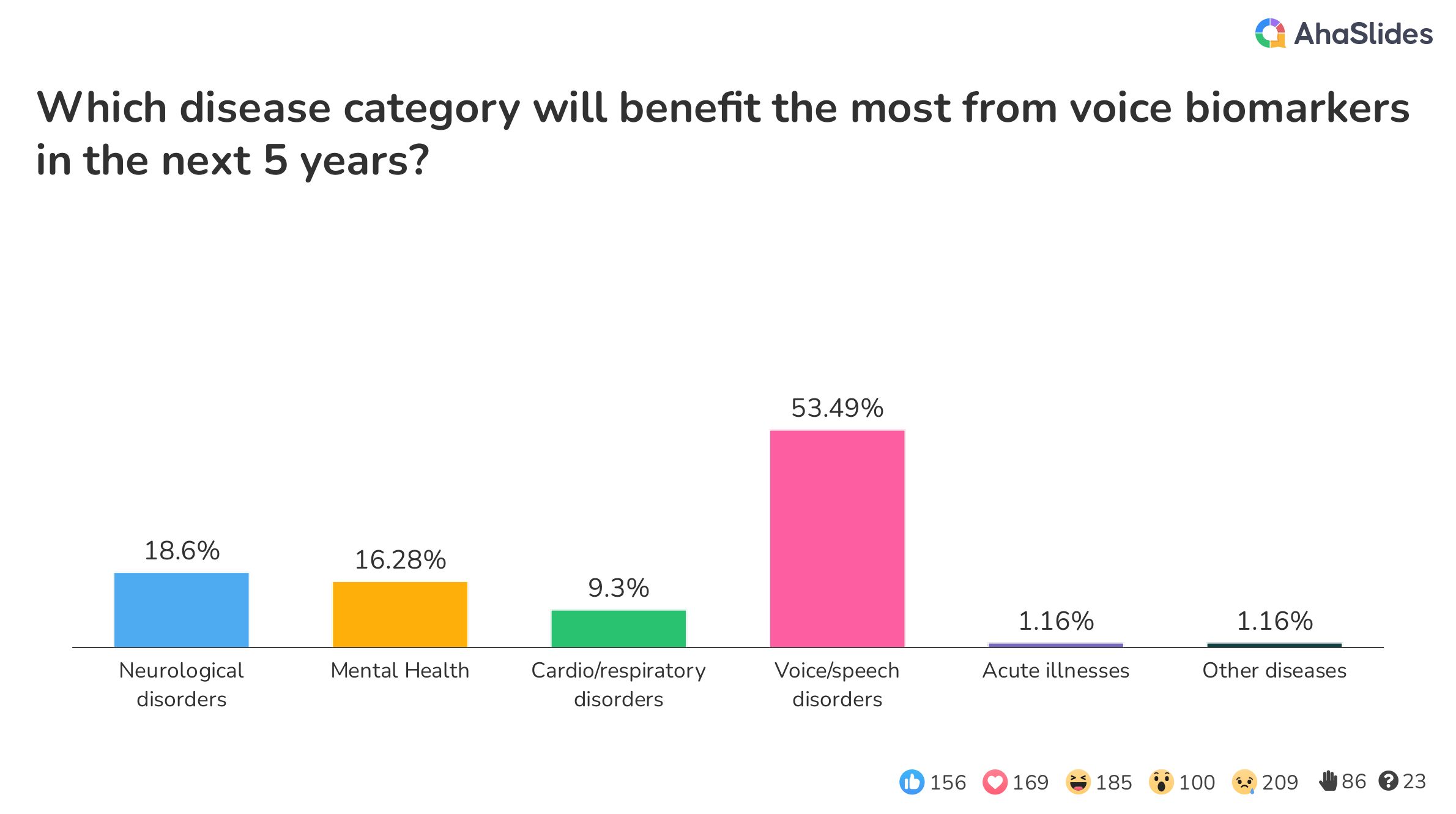

Supplement: Supplementary file 7 [file Image6.jpeg]

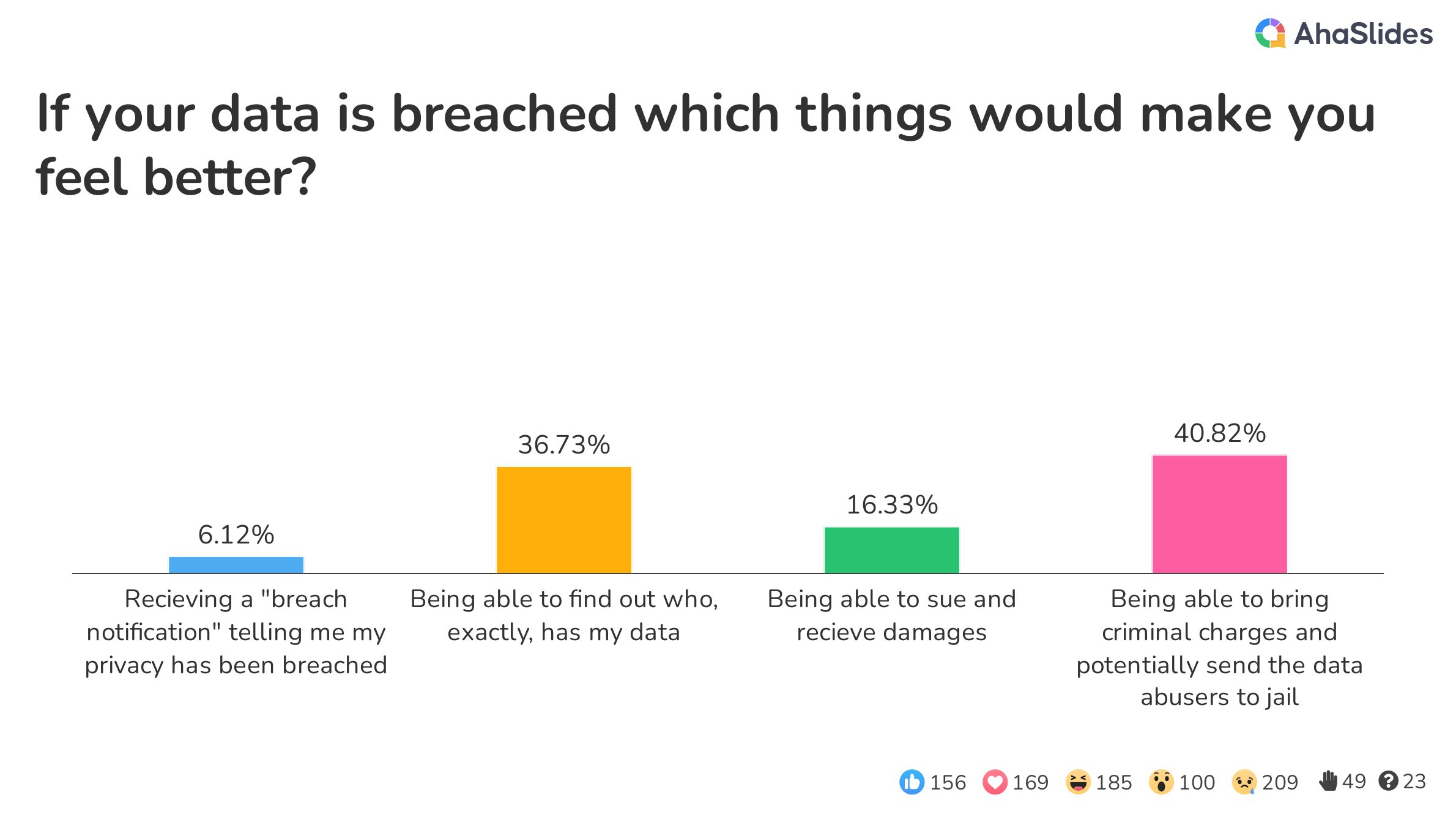

Supplement: Supplementary file 8 [file Image7.jpeg]

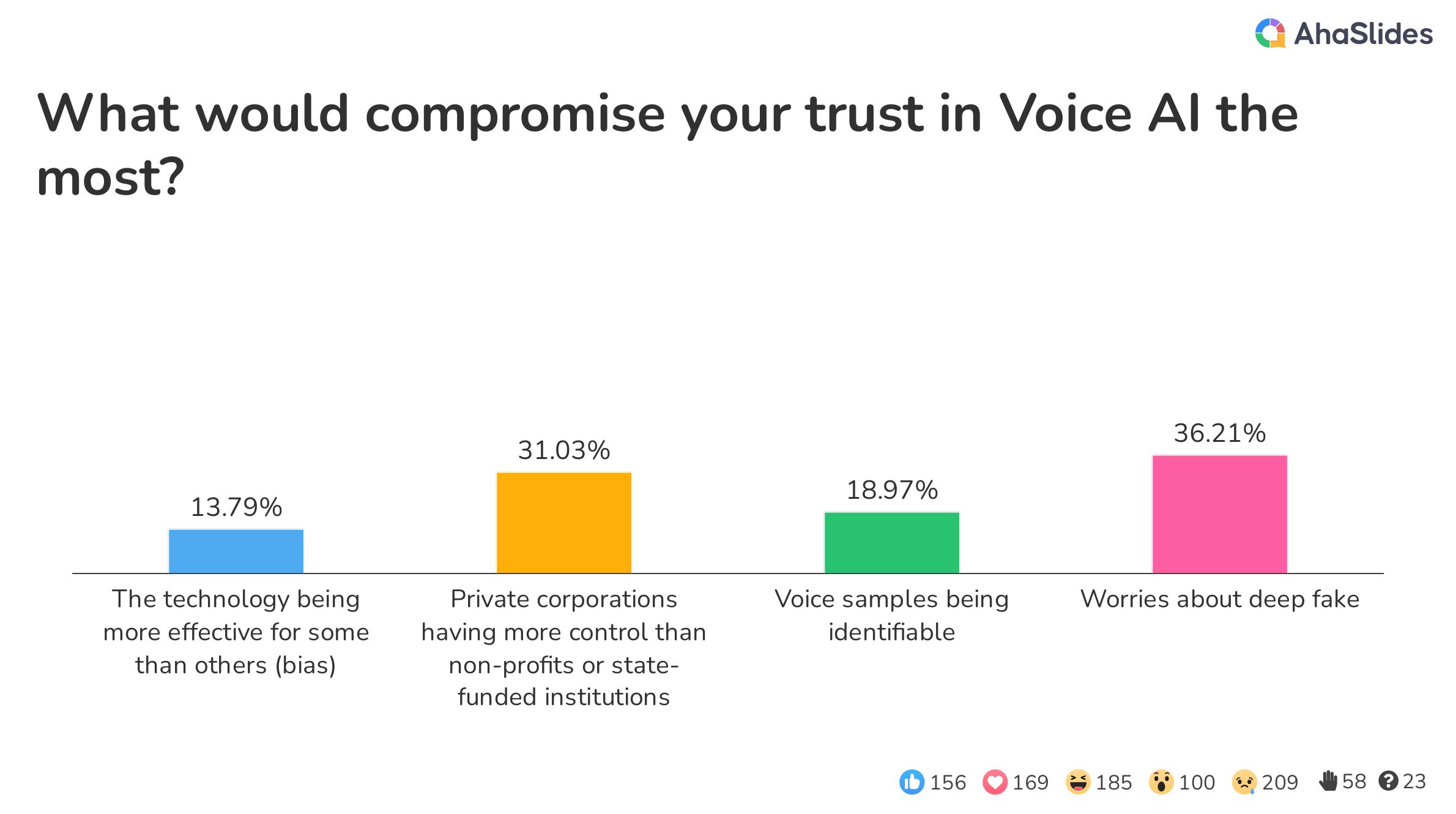

Supplement: Supplementary file 9 [file Image8.jpeg]

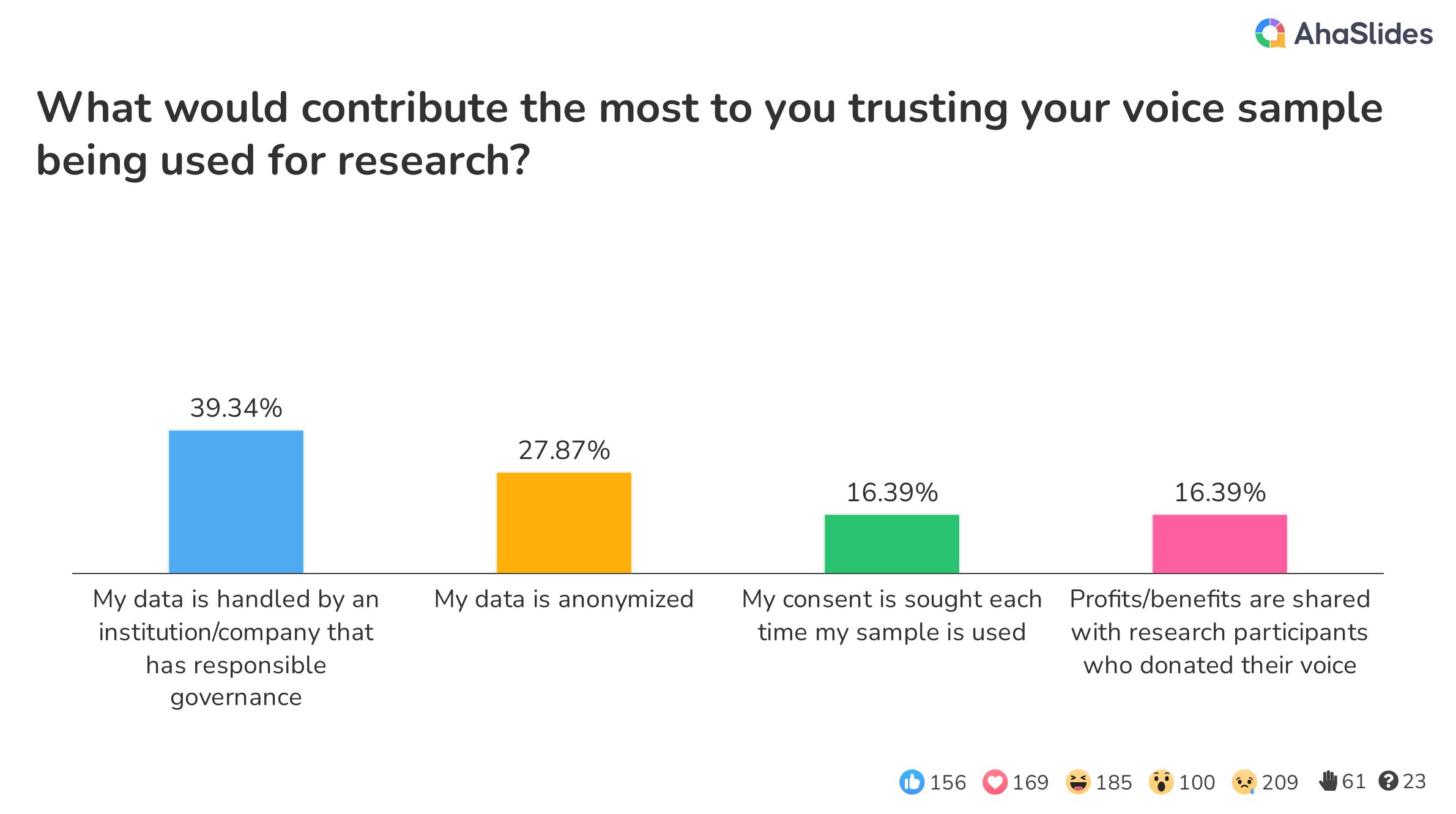

Supplement: Supplementary file 10 [file Image9.jpeg]

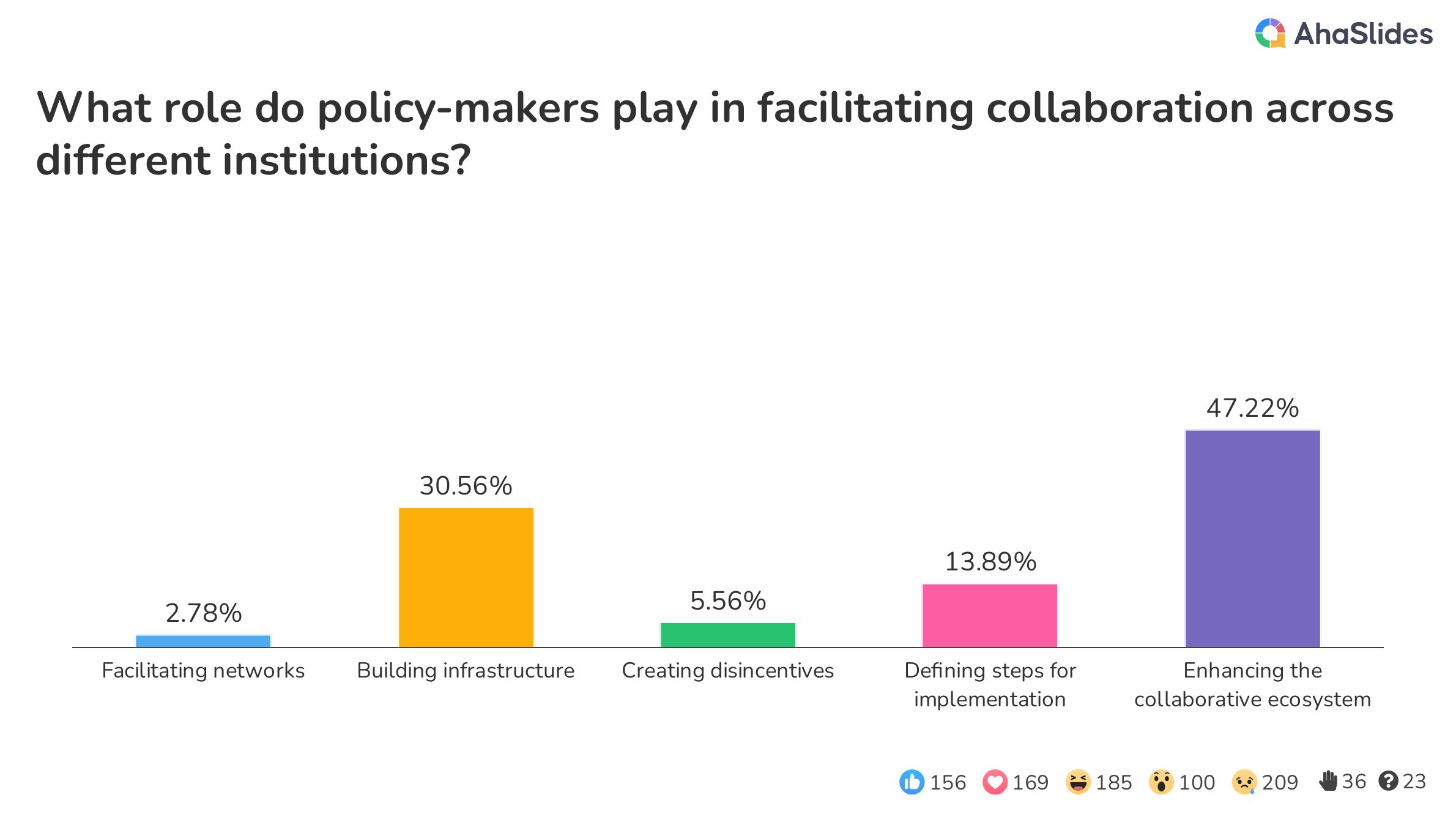

Supplement: Supplementary file 11 [file Image10.jpeg]

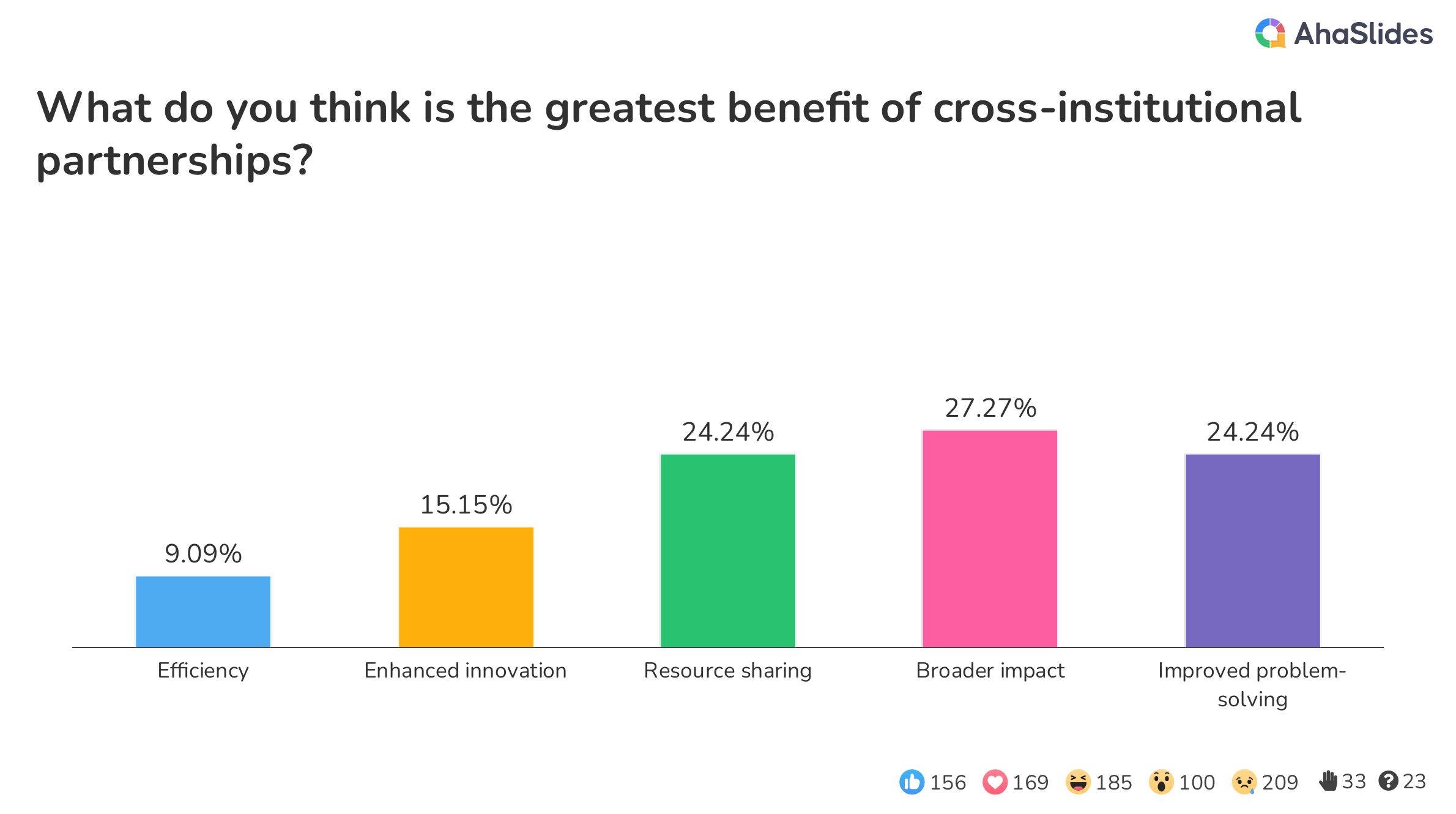

Supplement: Supplementary file 12 [file Image11.jpeg]
